# Participant Motivation Typologies as Correlates of Study Participation and Retention in Cognitive Aging Trials

**DOI:** 10.1093/geroni/igaf122.300

**Published:** 2025-12-31

**Authors:** Abigail Stephan, Hye Won Chai, Jody Nicholson, Christy Phillips, Lesley Ross

**Affiliations:** Clemson University, Clemson, South Carolina, United States; Clemson University, Clemson, South Carolina, United States; Clemson University, Clemson, South Carolina, United States; Clemson University, Clemson, South Carolina, United States; Clemson University, Clemson, South Carolina, United States

## Abstract

In recruiting and retaining participants in Alzheimer’s disease and related dementias (ADRD) research, participants’ motivations may explain why they participate in and withdraw from research. Drawing on Bardach and colleagues’ (2020) Activator, Motivator, Outside mediator, Re-enforcer (AMOR) model of research engagement, this presentation will report on our mixed-methods study that 1) explored participants’ reasons for participating and, in some cases, withdrawing from computerized cognitive training research studies and 2) connecting the provided reasons to study retention. A convenience sample of community-dwelling adults (N = 423) between 55-88 years (M = 67.45) was drawn from two randomized controlled trials involving computerized cognitive training. We used eclectic coding procedures to analyze participants’ reasons for participating (shared via written responses) and, when relevant, their stated reasons for withdrawing (ascertained via chart notes made by study personnel). Chi-square tests and logistic regression were used to test the associations between reasons for participation and study retention. Primary reasons for participating in research included activating and motivating factors, while primary reasons for withdrawing included outside contextual factors and re-enforcers. Re-enforcers were particularly relevant for withdrawal during the cognitive training phase. Reasons for participation were not associated with reasons for, nor likelihood of, withdrawal. This work suggests that individuals’ reasons for participating in and withdrawing from cognitive training studies are nuanced, with participants considering multiple factors in their decisions. Findings support an adapted version of the AMOR model for explaining research engagement, and further validation of a motivational research participation model is critical for identifying effective recruitment and retention efforts.

